# Use of Biologically Active 3D Matrix for Extensive Skin Defect Treatment in Veterinary Practice: Case Report

**DOI:** 10.3389/fvets.2019.00076

**Published:** 2019-03-15

**Authors:** Elena Yu Zakirova, Dmitry V. Shalimov, Ekaterina E. Garanina, Margarita N. Zhuravleva, Catrin S. Rutland, Albert A. Rizvanov

**Affiliations:** ^1^Department of Exploratory Research, Scientific and Educational Center of Pharmaceutics, Institute of Fundamental Medicine and Biology, Kazan Federal University, Kazan, Russia; ^2^Veterinary Clinic “6 Elephants”, Kazan, Russia; ^3^Faculty of Medicine, School of Veterinary Medicine and Science, University of Nottingham, Nottingham, United Kingdom

**Keywords:** mesenchymal stem cells, fibrin glue, 3D matrix, VEGF165, FGF2

## Abstract

**Objectives:** Large full-thickness skin defects represent a serious veterinary problem.

**Methods:** We have developed novel bioactive 3D-matrixes based on fibrin glue Tissucol (Baxter), containing the combination of the adenoviral constructs with genes vascular endothelial growth factor 165 (VEGF165) and fibroblast growth factor two (FGF2; construct Ad5-VEGF165 + Ad5-FGF2) or multipotent mesenchymal stem cells, genetically modified with these constructs.

**Results:** *In vitro* studies confirmed the biosynthesis of VEGF165 and FGF2 mRNA in the transduced cells. Ad5-VEGF165 + Ad5-FGF2- transduced multipotent mesenchymal stem cells showed an enhanced capacity to form capillary-like tubes *in vitro*. Bioactive 3D-matrix application enhanced granulation tissue formation and epithelialization of non-healing, large bite wounds in a dog. Successful wound healing was observed with a positive clinical outcome for the canine patient. This research and application of regenerative gene therapy alongside a novel bioactive 3D-matrix shows promising clinical applications for the future in both dogs and other mammals including humans.

## Introduction

Skin of pets such as dogs and cats is usually movable and relatively excessive. However, in veterinary practice, there are cases when it is impossible to close the skin defect with a simple apposition of wound edges (1). For example, this situation is observed in cases of large defects, wounds on distal parts of extremities or in oncology practice after radical resection of tumors. In veterinary surgery skin grafts, myocutanous flaps plastic surgery techniques are used for the closing of such skin defects (2). Unfortunately, in cases where extensive skin damage is a factor these methods are inapplicable. To treat such wounds we have developed, a bioactive 3D matrix based on fibrin glue, multipotent mesenchymal stem cells (MSCs), or/and *vegf165a* and *fgf2* genes containing adenoviral constructs and tested them both *in vitro* and in the veterinary clinic.

Healing of skin defects is a stepwise process, including hemostasis and inflammation in the early stages, followed by cellular proliferation and extracellular matrix deposition and ending with remodeling and scar formation (3). Wound healing requires the coordinated work of cells, growth factors, and extracellular matrix components. MSCs can influence all stages of wound healing process. They regulate immune responses and inflammation, and recruit macrophages, lymphocytes, and granulocytes to the inflammation area. MSCs also enhance migration and proliferation of keratinocytes and endothelial cells and secret growth factors [including vascular endothelial growth factor (VEGF), basic fibroblast growth factor/fibroblast growth factor 2 (bFGF/FGF2), epidermal growth factor (EGF), keratinocyte growth factor (KGF)] during proliferation phase of healing. MSCs can also differentiate into various lineages of mesenchymal origin and form dermal and hypodermal cell compartments (4, 5).

Angiogenesis is a key element of proliferative phase of wound healing because the delivery of oxygen and nutrients is crucial for reparation process. One of the most important proangiogenic factors is VEGF (6). VEGF stimulates DNA synthesis and proliferation and is involved in anti-apoptotic signaling pathways. It promotes proliferation and migration of endothelial and smooth muscle cells, monocytes, macrophages, and granulocytes involved in the wound healing process, stimulates the activity of pericytes and stabilizes newly formed vessels (7).

Recently it has been shown that VEGF stimulates wound healing not only due to its proangiogenic effects. *In vitro* studies have demonstrated that keratinocytes express both VEGF and its' receptors and VEGF directly stimulates their proliferation, migration and survival (8, 9). This factor stimulates migration of macrophages and cell debris uptake on early stages of wound healing and induces macrophage apoptosis during the resolution of inflammation (10, 11). FGF2 is a classic mitogen, which stimulates proliferation of target cells (including endothelial cells, fibroblasts, and keratinocytes), their migration and differentiation and also has a cytoprotective effect (12).

We developed a model of bioactive 3D-matrix based on fibrin glue Tissucol (Baxter), containing in one case MSCs, modified with a combination of adenoviral constructs with VEGF165 and FGF2 genes (MSC + Ad5-VEGF165 + Ad5-FGF2), and in the second case—the combination of the adenoviral constructs with genes VEGF165 and FGF2 (Ad5-VEGF165 + Ad5-FGF2) without MSCs. The aim of our study was to prove experimentally the feasibility and effectiveness of using a bioactive 3D-matrixes based on fibrin glue Tissucol (Baxter), containing the combination of the adenoviral constructs with genes VEGF165 and FGF2 (Ad5-VEGF165 + Ad5-FGF2) or MSC modified with the adenoviral constructs in a veterinary surgery setting for closing full-thickness skin defects in dogs.

## Materials and Methods

All procedures were performed in accordance with international, national, and/or institutional guidelines for the care and use of animals were followed. The Institutional Review Board of the Kazan Federal University approved this study (protocol No3; date 05.05.2015). A homeless dog, male, aged ~3–5 years, was in the custody of the animal protection community “Zoozabota” which took care of the animal and gave written informed consent. The procedure was only undertaken after conventional methods used to assist the dog had not helped recovery. The animal was too cachectic to harvest fat from. The mass of dog was 5 kg. During surgery the animal was kept anesthetized with 0.1–0.15 mg/kg Zoletil-100 (Tiletamine Hydrochloride, Zolazepam Hydrochloride, Virbac, France) and 1–3 mg/kg XylaVet (Xylazine Hydrochloride, Holland) under full veterinary care.

### MSC Isolation and Immunophenotyping

Due to the severity of the patient's condition we had to use allogenic dog adipose derived MSC for the generation of biologically active 3D matrix. MSCs were isolated using standard techniques as described previously (13). Briefly, the adipose tissue of donor dogs' greater omentum was isolated in a veterinary operating room during ovariohysterectomy under sterile conditions. The tissue was transported in a sterile flask with sterile 0.9% NaCl for further work in cell culture laboratory (within 2 h). The adipose tissue was cut to pieces of about 1 cm^2^ in a laminar flow biosafety cabinet under sterile conditions. Blood cells were washed out using centrifugation at 500 × g for 5 min. Adipose tissue was then incubated with the sterile solution of crab hepatopancreas collagenase (Biolot, Russia) to a final concentration of 0.2% for an hour at 37°C with shaking at 200 rpm. Next, the homogenate was centrifuged for 5 min at 500 × g, the enzymatic solution was then decanted and the cell pellet containing the stromal-vascular fraction was suspended in Dulbecco's phosphate-buffered saline (DPBS) solution and centrifuged twice for 5 min each at 500 × g to remove any residual enzymes. The obtained cells were cultured in α-MEM medium with 10% fetal bovine serum (FBS), 100 U/ml penicillin, 100 μg/ml streptomycin, and 2 mM L-glutamine (all from PanEco, Russia). The culture medium was changed every 3 days. Immunophenotyping of MSCs was performed using antibodies CD10 FITC, CD71 FITC (Sorbent, Russia), CD34 AF488, CD45 AF488, CD105 AF488 (Biolegend), CD44, stro-1, and Thy-1 (Santa Cruz, USA) according to manufacturers' protocols. Results were evaluated using a confocal fluorescence scanning microscope LSM 780 (ZEISS, Germany).

### Adenoviruses Ad5-VEGF165 and Ad5-FGF2 Preparation

Recombinant replication-defective adenoviruses Ad5-VEGF165 and Ad5-FGF2 were obtained using Gateway cloning technology as reported previously (14). Vegf165 gene cDNA fragments were amplified using thermocycler C1000 Thermo Cycler (BioRad), Phusion High fidelity DNA Polymerase (Finnzymes, Thermo Fisher Scientific, USA), and specific primers (Synthol, Russia). The purified PCR amplification products were cloned into a plasmid vector pENTR-D/TOPO (Invitrogen Thermo Fisher Scientific, USA) using topoisomerase followed by transformation into competent *E. coli* Top 10 cells. PCR screening of the colonies was carried out using vector-specific primers. Target recombinant plasmid uptake was confirmed by sequencing and restriction analysis.

PCR amplification of *fgf2* gene fragments was carried out in two stages. Initially attB—sites were connected using gene-specific primers hFGF2—attB1 and hFGF2—attB2 (15). The second stage was to increase the length of the nucleotide att-sites sequence which was performed using the adapter primer GW-attB1 and GW-attB2 (Liteh, Russia). BP- recombination was performed according to a standard protocol (Invitrogen Thermo Fisher Scientific, USA) followed by transformation into competent cells *E. coli* Top 10. PCR screening of colonies and target recombinant plasmid uptake confirmations were carried out as described above.

LR-recombination of gene cDNA from donor plasmids pENTR- VEGF165 and pDONR-FGF2 into vectors plasmid pAd/CMV/V5-Dest (Invitrogen Thermo Fisher Scientific, USA) was performed to create adenoviral expression constructs. Transformation of competent E. coli cells, the PCR screening of colonies and target recombinant plasmid receiving confirmation were carried out as described above.

To produce recombinant adenovirus Ad5-VEGF165 and Ad5—FGF2, adenoviral plasmid vector was transferred into a linear form using a restriction enzyme *Pac*I. HEK293A cells were transfected with purified linear plasmids using transfection reagent TurboFect. On the 10th day after transfection, fields of cytopathic effect became apparent and the cell suspension was collected. Cells were cryolysed, cell debris was eradicated using centrifugation to prepare a crude viral lysate. Amplification of recombinant adenoviruses Ad5-VEGF165 and Ad5—FGF2 was performed in HEK293A cell cultures. Adenoviruses were concentrated using ultracentrifugation in cesium chloride density gradient. Viruses were purified from cesium salts by dialysis. Determination of viral titers was performed by its capacity to form plaques on agarose layer.

### RT-PCR

Passage 3 MSCs were transfected with Ad5-VEGF165 and Ad5-FGF2 at 100 MOI each. The transcriptional activity of genes *vegf 165* and *fgf 2* were evaluated 24 h post-infection by qPCR. Specific primers, probes and nucleotide sequences were described previously (15). Serial dilutions of cDNA synthesized from mRNA of the transfected cells were used to construct a standard curve and determine the level of gene expression. The gene expression level of non-transfected cells was taken as 100%.

### Matrigel Tube-Formation Assay

Matrigel™ tube-formation assay with Ad5-VEGF165 and Ad5-FGF2 co-transfected cells and non-transfected (control) cells was performed as described previously (16). Briefly, 10,000 MSCs co-transfected with Ad5-VEGF165 and Ad5-FGF2 or 10,000 non-transfected cells per well in triplicates were seeded in a 96-well plate pre-coated with 50 μl of Matrigel^®^ Growth Factor Reduced Basement Membrane Matrix (Cat. #356231, Becton Dickinson, USA) in DMEM/F12 media supplemented with 1% FBS. The plates were incubated at 37°C in a humidified atmosphere containing 5% CO_2_ for 16 h. Tube formation was evaluated using microscopy and ImageJ software.

### Bioactive 3D Matrix Preparation

3D bioactive matrix was formed immediately prior to application to the wound. Fibrin glue Tissucol was used as a matrix basement. Two types of matrix were used. The first type of matrix consisted of 3,000,000 genetically modified MSCs and 1 ml of Tissucol. MSCs were co-transfected with Ad5-VEGF165 and Ad5-FGF2 at MOI 100 each the day before application. Cell suspension, thoroughly washed from media and viruses by centrifugation, was mixed with fibrin glue and uniformly applied to the wound surface. The second type of matrix was prepared from 1 ml of Tissucol and a mixture of Ad5-VEGF165 and Ad5-FGF2 viruses in amounts equivalent to those used for MSC transfection.

### Statistical Analysis

Statistical analysis was performed using Student's *t*-test in Microsoft Excel 2007 software, *P* ≤ 0.05 was considered statistically significant.

## Results

### *In vitro* Studies

**Figure 1:**
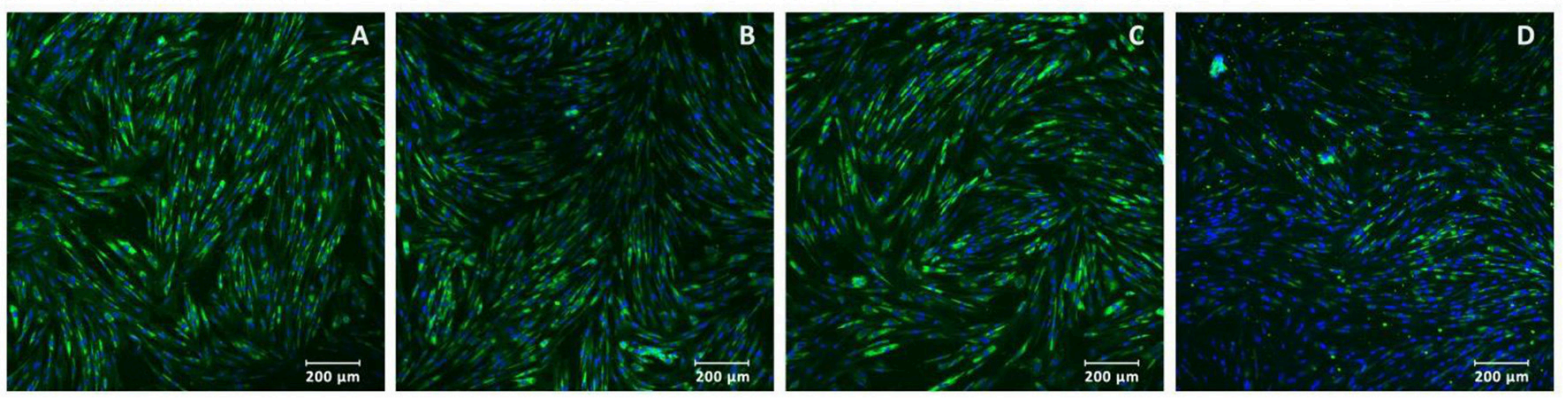
Immunocytochemistry of multipotent mesenchymal stem cells: **(A)** stro-1, **(B)** Thy-1, **(C)** CD10, and **(D)** CD105 expressed in green (488 wavelength). Nuclei were stained with DAPI (4′,6-diamidino-2-phenylindole, blue in color). Magnification: ×200.

Adenoviruses Ad5-VEGF165 and Ad5-FGF2 were prepared using standard methods of molecular genetics and used for gene therapy as part 3D-matrix or to modify the MSCs with MOI 100. MSCs isolated from dog adipose tissue as was described previously (17) had a fibroblast-like morphology, and expressed markers of the MSCs CD10, CD71, CD44, CD105, stro-1, Thy-1, and did not express the hematopoietic markers CD34 or CD45 (Figure 1).

**Figure 2:**
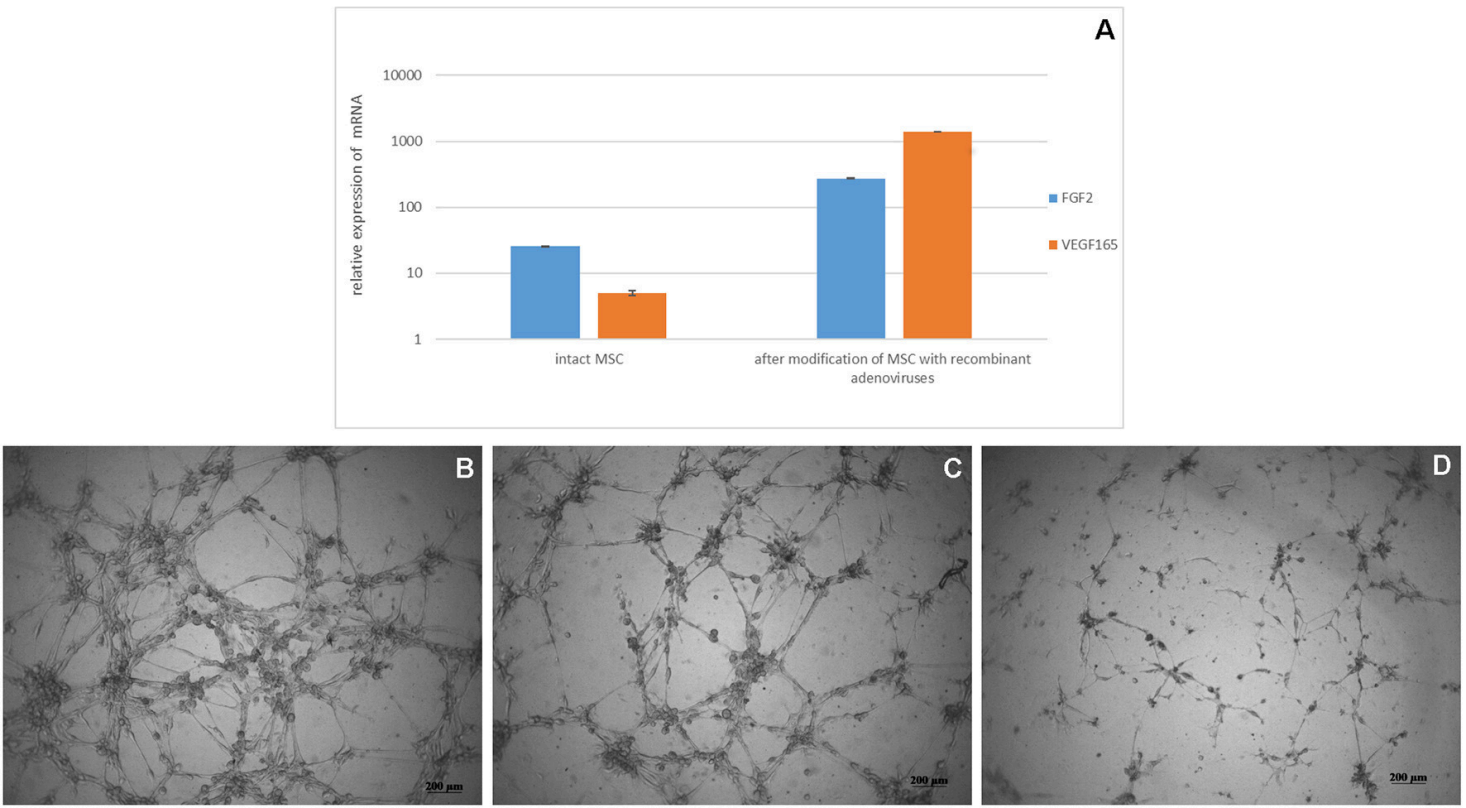
**(A)** Analysis of VEGF 165 and FGF2 mRNA expression in genetically modified MSCs. Data are presented as Mean ± standard error of the mean. Expression was normalized to 18s rRNA. Matrigel^TM^ capillary-like structure formation by genetically modified canine MSCs **(B)** Ad5-VEGF165 and Ad5-FGF2 co-transfected cells in DMEM/F12 media supplemented with 1% FBS **(C)** non-transfected cells on nutritious medium (positive control) in DMEM/F12 media supplemented with 1% FBS and 10 ng/ml recombinant VEGF, and **(D)** non-transected cells on poor medium (negative control) in DMEM/F12 media supplemented with 1% FBS.

Biosynthesis of VEGF and FGF2 mRNA in transduced cells was confirmed after 24 h after modification of MSC with recombinant adenoviruses (Figure 2A). MSCs-Ad-VEGF165-FGF2 had a higher (70 ± 5 units/well) capacity to form capillary-like structures on Matrigel^TM^ as compared with intact cells (47 ± 5 units/well, Figures 2A–D), *p* < 0.05.

### *In vivo* Clinical Case Results

**Figure 3:**
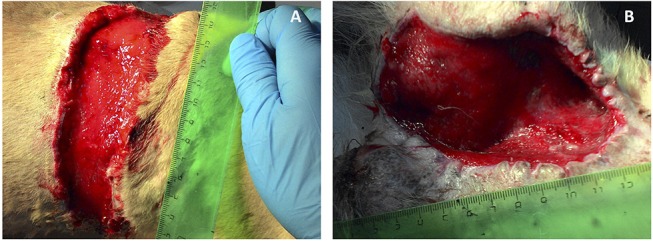
Canine wounds prior to 3D matrix application: **(A)** back, **(B)** groin.

*In vivo* biologically active 3D matrix was tested in two morphologically similar non-healing full-thickness bite wounds measuring about 70 cm^2^ each in the back and groin areas of a medium sized adult dog. The numerous bite wounds were received during fights between the patient and another dog. Immediately after injury, the wounds were sanitized and sutured in a veterinary clinic. The post-operative period proved complicated with extensive necrosis of the skin at the wound edges. Necrotic tissue was dissected away, as a result the closure of the wounds by matching their edges became impossible. The effect of standard practice conservative therapy was insignificant: after 2 weeks of treatment marginal necrosis was observed and granulations were almost absent. There was no wound contraction either. As the patient had not responded to standard veterinary care, treatment with the new therapy was administered. Necrotic skin was dissected and tissues were flayed until pinpoint bleeding (Figure 3).

In order to stimulate the regeneration of tissue growth around the wounds, the biologically active matrix was applied. The wound on the back was covered with matrix based on Tissucol fibrin glue containing 3 million MSCs transfected with combination of Ad5-VEGF165 and Ad5-FGF2 (MOI 100 for each construct). Allogenic MSCs had to be used due to the severity of the patients' body condition. The wound in the groin was covered with another type of matrix containing Tissucol fibrin glue, Ad5-VEGF165 and Ad5-FGF2 in amounts equivalent to those used to transduce the MSCs. Wounds were closed with occlusive dressings. To prevent wound contamination, Baytril 5% (enrofloxacin, Bayer Corporation, USA.) antibiotic was subcutaneously injected in dose 0.1 ml/kg once a day for 7 days.

**Figure 4:**
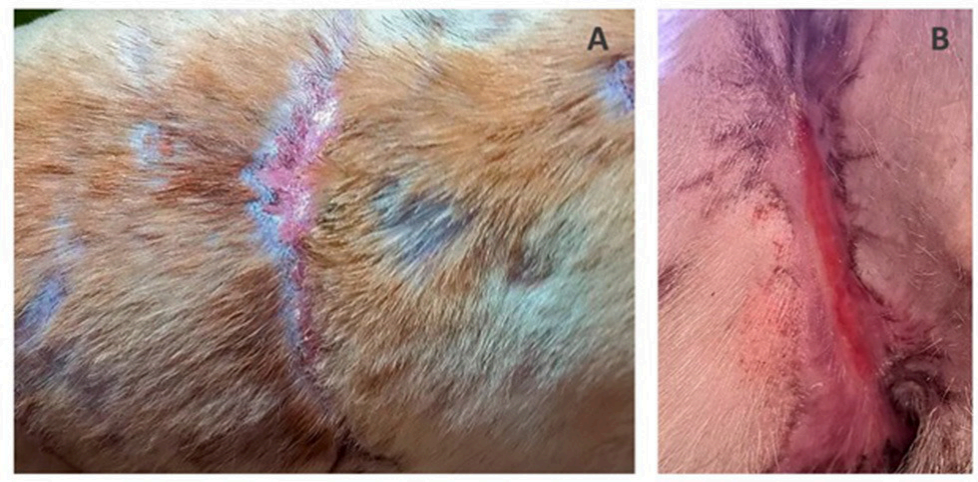
Canine wounds 1 month after 3D-matrix application: **(A)** back, **(B)** groin.

On the 3rd day after 3D matrix application the dressings were removed. Juicy red granulations, edge epithelization up to 3–4 mm, wound contraction and minor exudation were observed. On the 7th day, edge epithelization of from 6–7 to 10 mm had been achieved. There were no signs of necrosis or inflammation throughout the entire healing period. The wound on dogs' back was completely healed after 1 month and the wound in the groin healed within 1.5 months with normal non-hypertrophic scar formation (Figure 4). Following successful treatment the dog was adopted and rehomed as a pet.

## Discussion

Large full-thickness skin defects pose a serious medical and veterinary problem. In medicine if autodermoplasty is impossible, skin can be obtained from tissue bank. In veterinary medicine this is presently impossible. In addition large skin defects cause a vicious circle: the bigger the total lesion area, the worse the patients general condition, the worse local regeneration of damaged skin.

The effects of MSC, various growth factors and genetic constructs expressing them on the skin regeneration process have been studied for a long time. Positive effects of MSCs, VEGF, and FGF2 application on regeneration of skin defects in laboratory animals and in the clinic have been shown (18). Application of MSCs improves wound closure by accelerating epithelialization and increasing angiogenesis and granulation tissue formation (19, 20). The most beneficial effect of exogenous MSCs on wound healing was shown in cases of chronic wounds with poor trophicity (diabetic) (21, 22).

There is also evidence that VEGF and FGF2 can promote wound healing. VEGF is highly expressed in normally healing wounds but its excessive introduction doesn't significantly accelerate would closure and epithelialization (23, 24). However, in delayed healing diabetic wounds or ischemic skin flaps VEGF delivery enhances skin regeneration (25, 26). FGF2 is normally expressed in healthy skin an increased expression is observed upon skin damage. A number of studies have shown that FGF2 promotes tissue regeneration, and its deficiency causes wound healing disorders (27). Therefore, it has been suggested that FGF2 reduces hypertrophic scar formation (28).

There is a lack of studies of combined use of several growth factors together with a carrier matrix. Research has shown increased angiogenesis and keratinocyte growth after application of VEGF and FGF2-loaded collagen biomatrix on skin defects created on the back of fetal lambs (29). Our group has also had positive previous experiences of the use of genetic constructs containing the combination of VEGF and FGF2 genes in the treatment of chronic wounds (such as trophic ulcers in diabetes) (30) and injuries to tendons or ligaments in domestic animals (31, 32).

Our present study shows enhanced wound healing by combining the use of fibrin glue and MSCs, modified for increased production of VEGF165 and FGF2, or adenoviral constructs Ad5-VEGF165 and Ad5-FGF2. Our *in vitro* studies confirmed the biosynthesis of mRNA of vegf165 and fgf2 in the transduced MSCs. The resulting proteins VEGF and FGF2 exhibited their normal physiological activity. In addition they stimulated the formation of capillary-like tubes on Matrigel™, which is equivalent to the process of angiogenesis *in vivo*.

Upon application to wounds in a patient, satisfactory clinical results were achieved. Non-healing large bitten wounds were treated and enhanced granulation tissue formation, rapid epithelialization and wound contraction were observed and full recovery was achieved in a relatively short period of time. The difference in healing rate of two wounds (4 weeks for the back and 6 weeks for the groin) could be due to the location and shape of the wounds and/or MSCs secretion of the various biologically active substances which stimulated regeneration. These results highlight the need to further studies to investigate differing wound types in more patients as it shows great promise as a future treatment technique.

In conclusion the present clinical case has shown that fibrin glue based 3D matrices enhanced with Ad5-VEGF165 and Ad5-FGF2 or MSCs, transfected with Ad5-VEGF165 and Ad5-FGF2, have beneficial effect in the treatment of large non-healing wounds. This provides promising avenues of treatment for wounds and other conditions requiring tissue regeneration in animals.

## Author Contributions

EZ and AR contributed toward the conception and design of the study. All authors contributed toward data acquisition, analysis, and/or interpretation of data and drafted the work or revised it critically for intellectual content, and approved the submitted version. AR was awarded the funding.

### Conflict of Interest Statement

The authors declare that the research was conducted in the absence of any commercial or financial relationships that could be construed as a potential conflict of interest.
